# Leveraging Gene-Level Prediction as Informative Covariate in Hypothesis Weighting Improves Power for Rare Variant Association Studies

**DOI:** 10.3390/genes13020381

**Published:** 2022-02-19

**Authors:** Ying Ji, Rui Chen, Quan Wang, Qiang Wei, Ran Tao, Bingshan Li

**Affiliations:** 1 Department of Molecular Physiology & Biophysics, Vanderbilt University, Nashville, TN 37232, USA; Ying.ji@Vanderbilt.Edu (Y.J.); rui.chen.1@vanderbilt.edu (R.C.); quan.wang@Vanderbilt.Edu (Q.W.); qiang.wei@vanderbilt.edu (Q.W.); 2 Vanderbilt Genetics Institute, Vanderbilt University, Nashville, TN 37203, USA; 3 Department of Biostatistics, Vanderbilt University, Nashville, TN 37203, USA

**Keywords:** rare variant association study, gene prioritization, multiple hypothesis testing, false discovery rate, GWAS, machine learning, neuropsychiatric disorders

## Abstract

Gene-based rare variant association studies (RVASs) have low power due to the infrequency of rare variants and the large multiple testing burden. To correct for multiple testing, traditional false discovery rate (FDR) procedures which depend solely on P-values are often used. Recently, Independent Hypothesis Weighting (IHW) was developed to improve the detection power while maintaining FDR control by leveraging prior information for each hypothesis. Here, we present a framework to increase power of gene-based RVASs by incorporating prior information using IHW. We first build supervised machine learning models to assign each gene a prediction score that measures its disease risk, using the input of multiple biological features, fed with high-confidence risk genes and local background genes selected near GWAS significant loci as the training set. Then we use the prediction scores as covariates to prioritize RVAS results via IHW. We demonstrate the effectiveness of this framework through applications to RVASs in schizophrenia and autism spectrum disorder. We found sizeable improvements in the number of significant associations compared to traditional FDR approaches, and independent evidence supporting the relevance of the genes identified by our framework but not traditional FDR, demonstrating the potential of our framework to improve power of gene-based RVASs.

## 1. Introduction

Rare variant association studies (RVASs) enable the identification of disease-associated genes with clear functional support [1]. In RVASs, a large number of hypothesis tests are usually generated from scanning the human genome, and we need effective multiple-testing correction to limit false positives while maximizing power. False discovery rate (FDR) [2,3] control has become a popular approach for detecting weak effects by limiting the expected false discovery proportion (FDP). Of the FDR control procedures, the Benjamini Hochberg (BH) [2] procedure is one of the most commonly used. While BH is nearly optimal when all hypotheses are equally likely to be null [4], it suffers from suboptimal power when tests are heterogeneous [5], which is often the case in modern applications like RVASs.

Different from the BH procedure, hypothesis-weighting FDR control procedures have been proposed to incorporate prior information to up-weight or down-weight hypotheses [6]. The idea is that more FDR budget can be allocated to hypotheses with greater prior probability of being non-null, hence there is the potential to increase detection power [4,7].

We reason that hypothesis weighting might help improve detection power in RVASs. Previous research has shown the effectiveness of hypothesis weighting in multiple genetic applications, like differential expression gene discovery [8], GWAS [7,9,10,11,12], eQTL discovery [4,8], and trait-associated epigenetic marks discovery [13]. Recently, many hypothesis weighting methods have been developed, and a detailed comparison of these methods was reviewed elsewhere [14,15]. Among these methods, independent hypothesis weighting (IHW) [8] has been recommended due to its powerful, robust, and computationally efficient nature [13,14]. In addition, under the IHW framework, the relationship between covariates and *p*-values is assumed to be not fully known and can be learned as a function of the covariates from data without overfitting. This enables us to harness prior information that does not precisely specify but is informative of the likelihood of hypotheses being non-null. Leveraging these desired properties, we hypothesize that there might be opportunities to derive gene-level scores reflecting the prior likelihood of genes’ association with traits and use them as covariates in IHW method to increase detection power in RVASs.

Here, we propose a three-stage framework to improve the power of identifying risk genes from RVASs. Stage 1 is to identify a training set of risk genes and non-risk genes and build a prediction model using the training set and gene-level biological features; stage 2 is to generate gene-level scores for all genes in the genome using the previous built predictive models; stage 3 is to use the prediction scores as covariates to weight gene-level *p*-values from RVASs through the IHW method. Genome-wide association studies (GWASs) provide opportunities for us to derive gene-level scores to facilitate RVAS discoveries, as recent findings suggest the convergence of trait-associated genes from common and rare variants [16]. A key obstacle to leverage GWAS findings to improve the power of gene-based discovery is that GWAS loci are usually in noncoding regions, and implicating the risk genes for GWAS loci is a proven challenge [17,18]. As there are no clear sets of risk genes from GWAS, we employ integrative risk gene selector (iRIGS) [19] to classify genes near GWAS hits to high-confidence risk genes (HRG) and local background genes (LBG), and treat HRG and LBG as the positive and negative training sets in model building. We leverage the rich collection of gene-level annotations to identify patterns that are predictive of risk genes and use these annotations as biological features for model building and genome-wide prediction.

To demonstrate this framework, we propose to identify genes associated with Schizophrenia (SCZ) from gene-based *p*-values in a recent RVAS [16] using predictions informed from a recent GWAS [20]. As there is significant overlap of risk genes between SCZ and autism spectrum disorders (ASD) [16], we also propose to use the same predictions as covariates to adjust gene-based *p*-values in a recently published ASD RVAS [21].

## 2. Materials and Methods

### 2.1. Method Overview

**Figure 1:**
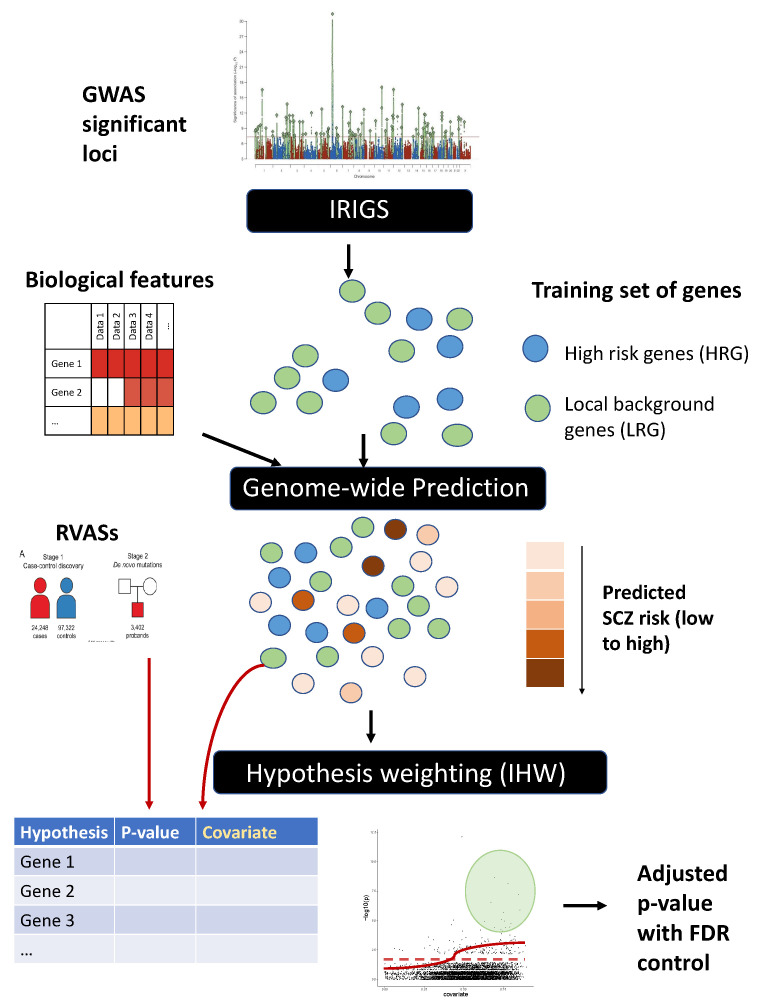
Overview of the workflow. Our approach involves three stages. First, we obtained HRGs and LRGs predicted by iRIGS as positive and negative instances for subsequent training; second, we used the positive and negative training sets and the selected gene-level biological annotations as features to train a Random Forest model, and used the model to generate predictive scores for all known protein-coding genes in the genome; third, we used the prediction score as the informative covariate for each gene, and used IHW to improve gene discovery from RVASs.

As shown in Figure 1, our approach involves three stages. First, we obtained HRGs and LBGs in 2 Mb windows centered at SCZ GWAS significant loci using iRIGS [19]. The two sets of genes (i.e., HRG, LBG) identified via iRIGS served as positive and negative instances for the subsequent training. Second, we used the positive and negative training sets and the selected gene-level biological annotations as features to train a Random Forest model [22], and used the model to generate predictive scores for all known protein-coding genes in the genome. Third, we used the prediction score as the informative covariate for each gene, and used IHW to improve gene discovery from RVASs. Details of the three steps are described below.

### 2.2. Obtain Training Set of Genes

We need a training set of “SCZ-genes” and “non SCZ-genes” to build a supervised machine learning model. Here, we used iRIGS to obtain these genes. All genes within a 2 Mb region centered at the previously reported SCZ GWAS significant index SNPs are considered as candidates [20]. The genes with the highest iRIGS posterior probability (i.e., HRG) from all the GWAS hits are used as “positive” instances, and the genes with posterior probabilities less than median of all candidate genes (i.e., LBGs) are used as “negative” instances.

### 2.3. Feature Pre-Processing

BRAINSPAN [23] is a dataset with RNA sequencing profiling with different cortical and subcortical structures across the full course of human brain development. The dataset includes 524 samples with developmental time points ranging from 5 post-conceptional weeks (pcw) to over 40 years of age from 26 brain structures. We used the gene expression values measured across different developmental time points and brain structure as the feature for training.

DEPICT [24] provides a dataset with 14,461 “reconstituted” gene sets with membership probability for each gene in each gene set based on co-regulation of gene expression and previously annotated gene sets representing a wide specturm of biological annotations. We used the genes and their membership probabilities across the 14,461 reconstituted gene sets as the features for training.

FANTOM5 [25] project used cap analysis of gene expression (CAGE) to measure promoter utilization across 975 human samples. We used the genes’ CAGE expression values as feature for training.

LAKE [26] dataset includes nuclear transcriptomic data for >60,000 single cells from human adult visual cortex, frontal cortex, and cerebellum from six different individuals. For each gene, we take an average of all the expression in all cell types and states (with labels provided in the dataset) to reduce the dimension of this dataset. We constructed a matrix with 61 columns, and use this matrix as features for training (dataset is made available at https://github.com/yingji15/SCZIHW_public/blob/main/data/features/all.count.region.allcommgeneid, accessed on 1 May 2020).

### 2.4. Model Training and Genome-Wide Prediction of SCZ Risk

We seek to prioritize SCZ risk genes using machine learning approaches; in this study, we employed random forest classifier. We used HRGs and LBGs predicted by iRIGS as positive and negative instances (i.e., genes for training), and gene-level genomic annotations predictive of SCZ risk genes as features. For genomic features, we used gene expression data from BRAINSPAN, DEPICT, FANTOM5, and LAKE, as described previously. As there are usually one HRG and multiple LBGs from each significant locus, the training set is highly imbalanced. To tackle this issue, we downsampled [27] the negative genes to create a balanced training set of the same number of positive and negative genes for each iteration. In each iteration, we performed a three-fold cross-validation [28] to tune the random forest classifier. That is, we randomly divided the downsampled dataset into three folds of approximately equal sizes. Each time, we fitted the model using two folds and then validate its prediction performance in the remaining fold. We chose the best random forest classifier based on the average performance across three validation folds, and then used this classifier to make prediction of all genes in the genome. We repeated the process 100 times, and take the average of the prediction scores as the final score for each gene. We used the R package “randomForest” [29] for the implementation and the only parameter we set is the number of trees (ntree = 3000).

### 2.5. Application of IHW for Hypothesis Weighting

IHW is a general method with established type I error control and stability. Intuitively, individual tests may differ in their statistical properties and a covariate might provide information for such properties. For our case of gene-level rare variant association hypotheses, genes may differ in their relevance to SCZ risk, and the level of their relevance is reflected by their prediction scores from the random forest model; this relevance can be indexed by gene-level covariates. Then, instead of using a flat *p*-value threshold in conventional methods, we can use an adaptive threshold informed by the covariate: Allocate more FDR budget to hypotheses with higher covariate values.

To explain the methods, suppose we have *m* hypotheses to test based on *p*-values ($p_{1},\ldots ,p_{m}$) with covariates $X_{1},\ldots ,X_{m}$. Conventional BH-approach uses this decision rule:

(1) $$\mathrm{Reject}\;\mathrm{hypothesis}\;i\;\mathrm{if}\;p_{i}\leq \hat{t},$$

with cutoff $\hat{t}$ determined at a defined level using only *p*-values by a multiple testing procedure family-wise error rate (FWER) control or FDR control, such as Bonferroni correction [30] or BH [2] respectively, to protect against spurious discoveries.

Instead of using the conventional approach illustrated in Equation (1), we used “IHW” [8,15], a general and flexible hypothesis weighting approach unique in that it can learn weights from covariates and *p*-values without overfitting (i.e., losing type-I error control) using cross-weighting. Under IHW, a decision rule is:

(2) $$\mathrm{Reject}\;\mathrm{hypothesis}\;i\;\mathrm{if}\;p_{i}\leq \hat{t}\hat{W(X_{i}^{-l})}\;\mathrm{where}\;i\in I_{l},$$

where $I_{l}$, l=1,…,k is a partition of the hypotheses into *k* folds to avoid overfitting. $\hat{W(X_{i}^{-l})}$ are weight functions depend on covariates, with the weight function used for fold *l* learned from *p*-values and covariates *X* from the k−1 folds excluding the fold *l*. Compare Equation (2) and Equation (1), it is equivalent to using weighted *p*-values ($p_{i}/\hat{W(X_{i}^{-l})}$) instead of *p*-values ($p_{i}$) in multiple hypothesis testing. The genes with large weights yield smaller weighted *p*-values, and the associated genes are more likely to be declared significant. Here, IHW splits the hypotheses into different strata (selected using the default mode “auto”) based on increasing value of the predicted gene-level risk score. Within each stratum, IHW randomly split them into folds. IHW learns the weights for each stratum and fold combination to achieve the highest number of discoveries. Details of IHW can be found in [8,15].

### 2.6. The SCZ RVAS Data

Gene-level association *p*-values for SCZ RVAS were obtained from the Schizophrenia Exome Sequencing Meta-analysis (SCHEMA) consortium [16] (https://schema.broadinstitute.org/results, accessed on 1 May 2020). The data contain the meta-analysis of whole-exomes of 24,248 cases and 97,322 controls from diverse global populations. Three classes of ultra-rare (defined as minor allele count ≤ 5) variants are included in the meta-analysis: Protein-truncating variants PTVs (defined as stop-gain/loss, frameshift, essential splice donor and acceptor variants), missense variants with missense badness, PolyPhen-2, and constraint (MPC) pathogenicity score > 3, and missense variants with MPC pathogenicity score = 2 or 3. PTVs and MPC > 3 variants (defined as “class I” variants in [16]) were analyzed by a burden test to generate gene-level *p*-values; genes with MPC = 2 or 3 variants were aggregated and combined with class I *p*-values using a weighted Z-score method, refer to Singh et al. [16] for details. We extracted the meta-analysis *p*-values (column “P_meta” in the online table) for the analysis.

### 2.7. The ASD RVAS Data

Gene-level association test results for ASD RVAS were obtained from Supplementary Table S2 of Satterstrom et al. [21]. FDR q-values are transformed to *p*-values for analysis (code is available at https://github.com/yingji15/SCZIHW_public/, accessed on 1 May 2020). The data contain the largest exome sequencing study of ASD to date (n = 35,584 total samples, 11,986 with ASD). Two categories of rare variation (allele frequency ≤0.1%), namely protein-truncating variants (PTVs; i.e., frameshift, stop gain/loss, canonical splice site disruption) and “probably damaging” missense variants according to PolyPhen-2 (Mis3) [31], are included in the analysis, with association signals from three categories of sources: De novo mutations, inherited variants in parent-offspring trios, and case-control association analysis.

## 3. Results

### 3.1. Evaluation of Prediction Scores

**Figure 2:**
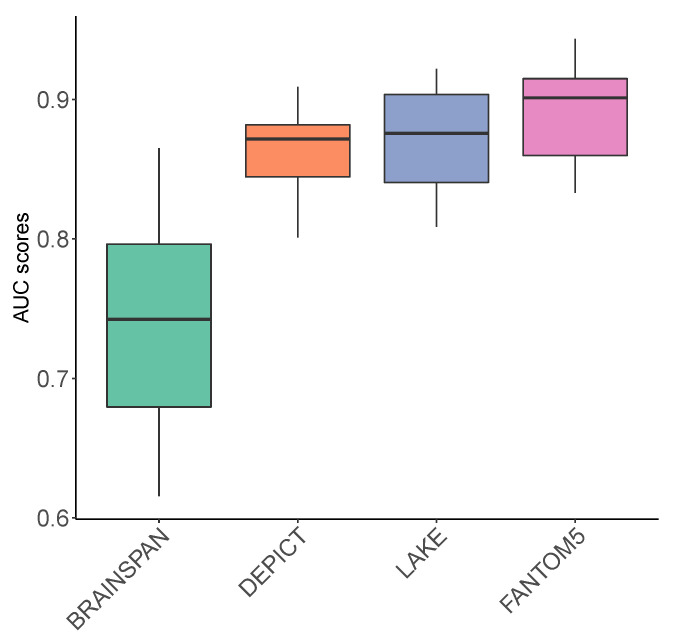
Distribution of AUC scores across the predictions from different features. The average AUC of 0.74, 0.86, 0.87, and 0.89 were obtained from BRAINSPAN, DEPICT, LAKE, and FANTOM5, respectively.

As shown in Figure 2, we first evaluated our prediction models using cross-validation and the model achieved an average area under the receiver–operator curve (AUC) of 0.74, 0.86, 0.87, and 0.89 using features obtained from BRAINSPAN, DEPICT, LAKE, FANTOM5 respectively. Among the selected features, DEPICT, LAKE and FANTOM5 showed comparable performance in terms of AUC, while BRAINSPAN based prediction showed lower AUC compared to the other three. The AUC values are all higher than 0.5, demonstrating that they all contain informative signals about SCZ risk.

**Figure 3:**
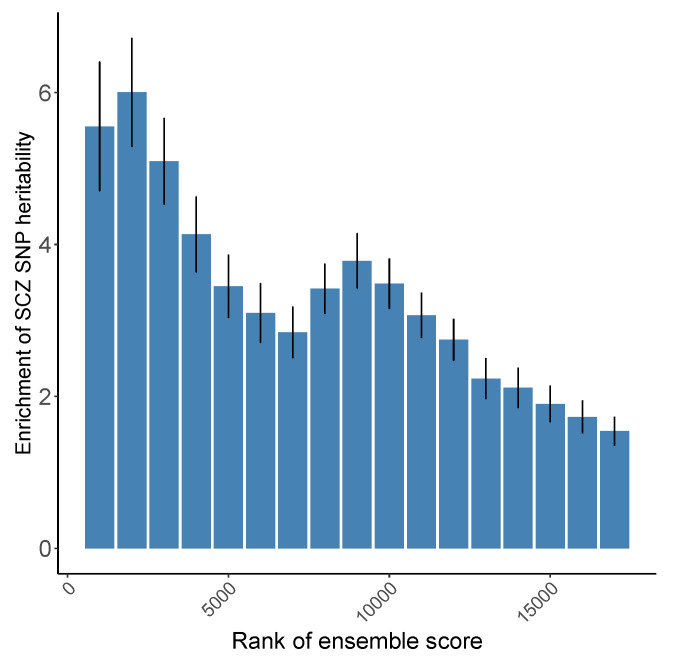
Enrichment of schizophrenia-SNP heritability with the number of ranked genes (calculated using LDSC). The most recent SCZ GWAS results published in 2020 [21] was used in the analysis. Top ranked genes are significantly enriched for SNP-based heritability.

Since different features may characterize SCZ risk genes from different angles, we generated an “ensemble score” as the final gene prediction by averaging the min-max normalized predictions from individual features: For each feature, the minimum value of the prediction gets transformed into a 0, the maximum value gets transformed into a 1, and every other value gets transformed into a decimal between 0 and 1. Then we performed a systematic empirical evaluation based on the enrichment of SNP-based heritability by stratified LD score regression (LDSC) [32,33] according to the ranking of the ensemble score. As shown in Figure 3, we found that the top ranked genes are significantly enriched for SNP-based heritability through applying LDSC on a most recent SCZ GWAS [34].

We further evaluated the ensemble score-based gene ranking using enrichment analyses with gene lists repeatedly implicated in SCZ [19]. As shown in Table 1, we evaluated the top 1000 predicted genes by ensemble score versus the rest of the genome for enrichment using one-sided Fisher’s exact tests. We found strong enrichment in FMRP target genes ($p=6.10\times 10^{-249}$), which is an RNA-binding protein that regulates translation and needed at synapses for glutamate receptor signaling and neurogenesis [35,36]. We also found top predicted genes to be significantly enriched in synaptic genes, including postsynaptic density (PSD, $p=5.82\times 10^{-126}$), protein cytoskeleton-associated scaffold protein (ARC, $p=2.19\times 10^{-8}$), NMDAR network ($p=3.54\times 10^{-24}$), mGluR5 ($p=2\times 10^{-5}$). We also observed significant enrichment in RFBOX1 ($p=2.26\times 10^{-140}$) and miR-137 targets ($p=2.19\times 10^{-22}$). A detailed description of the gene lists is in (Appendix A) Table A1.

### 3.2. Leverage Prediction as Covariates to Identify SCZ Risk Genes

Having evaluated our predicted scores using different evidence, we sought to examine the utility of the predictions for the identification of risk genes from RVAS results. Here, we extracted the gene-based association *p*-values from SCZ RVAS [16] and investigated the ensemble scores as covariates to conduct hypothesis weighting in IHW.

**Figure 4:**
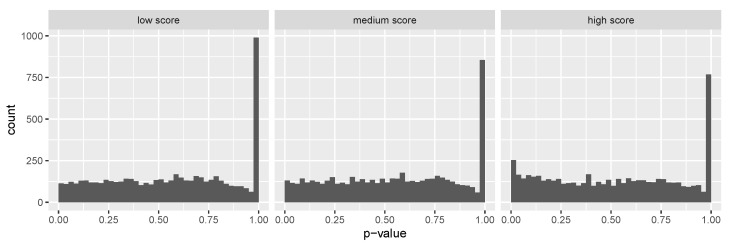
Histograms of SCHEMA reported [16] *p*-values after splitting the hypotheses into three groups by the prediction score. A successive increase of the number of hypotheses with *p*-values near zero is observed for increasing scores, indicating that the proportion of non-null effects varies across different groups.

As an exploratory analysis, we first checked whether the ensemble score as a covariate is informative about power under the alternative. We started with SCZ RVAS results by partitioning all hypotheses into three equally sized groups: “Low score” group with the ensemble score less than its 33% quantile, “medium score” group with the ensemble score between its 33% and 67% quantile, and “high score” group with the ensemble score larger than its 67% quantile. As shown in Figure 4, we observe a successive increase of the number of hypotheses with *p*-values near zero for increasing scores, indicating that the proportion of non-null effects vary across different groups.

Since the ensemble scores are informative of the prior probability of each individual test, to maximize power for discovery, all gene-level tests should not be treated exchangeably. Thus, we used the ensemble scores as covariates to adjust RVAS gene-level *p*-values under different target FDR levels (α = 0.05, 0.1, 0.2, 0.3) using IHW. The range of α is chosen to reflect the FDR control level commonly used in practice. We also included the prediction scores from individual feature sets (i.e., BRAINSPAN, DEPICT, LAKE, FANTOM5) as covariates for comparison purpose. As shown in Table 2, when using the ensemble score as the covariate to adjust SCZ *p*-values, although we did not find an increase of significant genes when α=0.05 potentially due to insufficient power, we did observe 22%, 28% and 109% increase of significant genes for higher target FDR levels at α=0.1,0.2,0.3 respectively. For the single feature based scores, we observe more improvement from DEPICT and LAKE data, and less improvement from BRAINSPAN and FANTOM5 (Table 2). It is evident that the ensemble score-based approach is able to identify more genes than individual features-derived scores (Table 2).

One might doubt whether the power gain is just by chance. To check this, we randomly shuffled the ensemble scores (i.e., “IHW-shuffled ensemble” in Table 2), and used the shuffled scores as the covariate for adjustment. The number of rejections is similar to that using the BH approach, providing evidence that a mis-specified covariate would not cause power increase or decrease. This result is in line with the previous findings that hypothesis weighting can lead to power improvements with informative weights and cause little power loss with uninformative weights [6,25,37].

**Figure 5:**
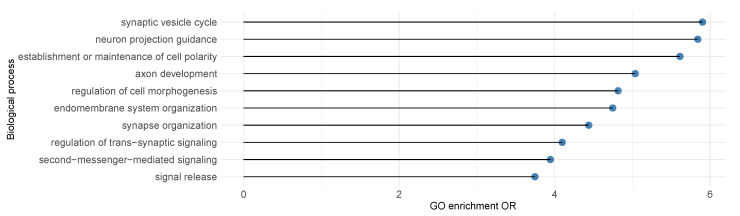
SCZ gene ontology(GO) enrichement analysis results for top biological processes with FDR ≤0.1. Enrichmed biological processes including synapse assembly (OR = 5.90) and neuron projection guidance (OR = 5.84) were discovered at FDR = 0.1, consistent with previous knowledge on SCZ.

Using the expanded set of significant genes identified, we next sought to gain more biological insights. We applied gene ontology (GO) enrichment analysis to the 84 genes that are insignificant using BH-adjustment but significant after IHW-adjustement at FDR level α=0.3. As shown in Figure 5, we discovered enrichmed biological processes at FDR = 0.1 including synapse assembly (OR = 5.90), neuron projection guidance (OR = 5.84), consistent with current knowledge on SCZ [38]. We also evaluated the 64 genes that are significant under the conventional BH-adjustment at α=0.3 for GO term enrichment and observed none of the GO terms are significant at FDR = 0.1 level.

Then we further investigated genes not significant using BH-adjustment but “boosted” to significance after adjustment using the ensemble score in IHW (referred to as IHW adjustment). The FDR level α=0.1 is chosen since that’s the significance level at which the adjustment leads to improvements. Since the RVAS study we used is comprehensive and included most available RVAS studies, we were not able to conduct replication studies. Instead, we looked for literature support for genes “boosted”. CACNA2D1 is one example, not significant under traditional BH-adjustment (p=0.23) but significant after IHW adjustment (multiple-testing adjusted p=0.065). A deletion in CACNA2D1 has been observed in one Japanese SCZ patient from in an independent study [39]. There is also support for CACNA2D1 in other psychiatric disorders that are correlated with SCZ, e.g., epilepsy and intellectual disability [40], and CACNA2D1 has been identified as a potential drug target in MDD from GWAS [41]. Another example is FABP7, not significant under traditional BH-adjustment (p=0.21) but significant after IHW adjustment (p=0.065), in which non-synonymous variants have been identified associated with SCZ and ASD [42]. FYN not significant under traditional BH-adjustment (p=0.17) but significant after IHW adjustment (p=0.045), harbors an excess of disruptive and damaging variants among SCZ patients [43]. For all these genes, the ensemble score derived in our framework reflects true association signals and its use as the covariate in IHW provides extra confidence that these genes are likely be SCZ risk genes.

### 3.3. Leverage Prediction as Covariates to Identify ASD Risk Genes

**Figure 6:**
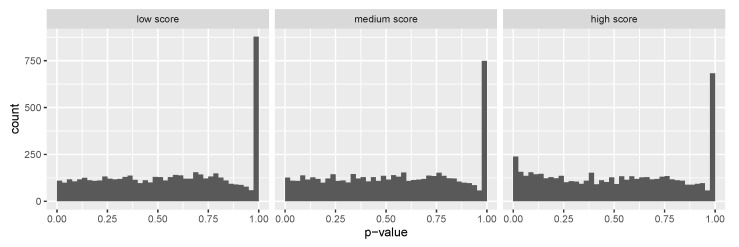
Histograms of ASD *p*-values after splitting the hypotheses into three groups by the prediction score. A successive increase of the number of hypotheses with *p*-values near zero is observed for increasing scores, indicating that the proportion of non-null effects varies across different groups.

As another application, we sought to evaluate whether our ensemble score can serve as covariates for the detection of risk genes from RVAS in ASD. While the prediction scores were derived using SCZ risk genes, multiple lines of evidence have suggested that SCZ and ASD share underlying genetic mechanisms: SCZ and ASD are genetically correlated [44]; up to 30% of individuals diagnosed with ASD during childhood will develop SCZ in adulthood [45]; CNVs and rare alleles show overlap between ASD and SCZ in synaptic related genes [46,47]. Thus, we used the same SCZ derived scores as covariates in IHW to adjust *p*-values from the largest RVAS of ASD [21]. Exploratory plots in Figure 6 suggest that our ensemble score is also informative in stratifying ASD test results.

As shown in Table 3, when using the ensemble score as the covariate, we observe 47%, 77%, 125% and 230% increase of significant genes for α=0.05,0.1,0.2,0.3 respectively, showing a similar trend of increased association detection after adjustment as in SCZ. For the single feature based scores, we observe more improvement from BRAINSPAN, DEPICT and LAKE data, and less improvement from FANTOM5 (Table 3). These findings are consistent with the overlapping genetic basis in SCZ and ASD.

**Figure 7:**
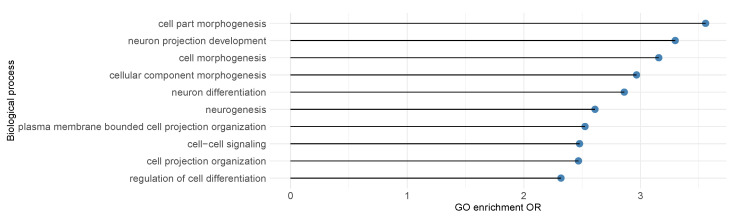
ASD gene ontology(GO) enrichement analysis results for top 10 biological processes with FDR ≤0.05. Enrichment of biological processes including cell part morphogenesis (OR = 3.56), neuron projection development (OR = 3.29), and neuron differentiation (OR = 2.86) were discovered at FDR = 0.1, consistent with current knowledge on ASD.

Similar to previous analysis on SCZ, using the expanded set of significant genes identified, we next sought biological insights. We applied gene ontology enrichment analysis to the 488 genes that are insignificant using BH-adjustment but significant after IHW-adjustement at FDR level α=0.3. As shown in Figure 7, we discovered the enrichment of biological processes like cell part morphogenesis (OR = 3.56), neuron projection development (OR = 3.29), and neuron differentiation (OR = 2.86), consistent with previous knowledge on ASD [48].

Then we further investigated genes not significant using BH-adjustment but “boosted” to significance after adjustment using the ensemble score in IHW (referred to as IHW adjustment). At FDR level α=0.1, 84 genes are “boosted” by IHW adjustment. For the same reason as SCZ, we were not able to find independent RVAS studies for replication. We looked for literature support for genes “boosted”. COBL is not significant under traditional BH-adjustment (*p*-value = 0.37) but significant after IHW adjustment (*p*-value = 0.095). It has been shown that deletions of COBL cause defects in neuronal cytoskeleton morphogenesis in model vertebrates [49]. It has also been supported by case-unique CNVs in autism case-control studies [50]. GABRA1 is not significant under traditional BH-adjustment (p=0.34) but significant after IHW adjustment (p=0.086). Previous studies have found significant reductions of GABRA1 expression in several brain regions for subjects with ASD [51].

## 4. Discussion

In this study, we explored the use of IHW in analyzing RVAS results with gene-level predicted scores as covariates, and investigated the implicated risk genes in biology of SCZ and ASD. The covariate is the predicted gene-level susceptibility to SCZ obtained through supervised learning using biological features from BRAINSPAN, FANTOM5, DEPICT and LAKE as inputs. An ensemble score, which is the average of all single-feature-based predictions, is derived to capture support from all features. Applications of the ensemble score in IHW to gene-level *p*-values in SCZ and ASD RVASs lead to more significant genes than traditional methods, suggesting the benefits of integrating diverse biological evidence. This is consistent with previous findings that integrating multiomics covariates improves power in identifying SNPs from GWAS analysis and eGenes from eQTL analysis [11]. In particular, when using the ensemble scores as covariates, we observed 22%, 28% and 109% more significant genes for target FDR levels at α=0.1,0.2,0.3 respectively for SCZ RVAS analysis; and 47%, 77%, 125% and 230% more significant genes for α=0.05,0.1,0.2,0.3 respectively for ASD RVAS analysis. We have identified genes not significant using BH-adjustment but “boosted” to significance after adjustment using the ensemble score in IHW in both SCZ and ASD with literature support, demonstrating the power of the proposed approach.

Previous studies have shown that the hypothesis weighting adjustment mostly has an impact on the genes with “borderline significance”. Genes with very small *p*-values already have high power, and conversely, genes with very large *p*-values have extremely low power and benefit little by weighting. Therefore, the weighting approach is most useful for genes with a marginal effect [6]. Here, for SCZ, we observe more improvement at FDR > 0.1, and the reason might be that there are more genes at the borderline at FDR > 0.1 yet very few genes are at the borderline when FDR = 0.05. On the other hand, for ASD, we observe improvements across different FDR levels, suggesting there are more borderline genes at each FDR level. This might come from the larger power of rare-variant gene-level tests in ASD.

There are a few limitations in our framework. First, the training set in the prediction scoring process is from genes inferred by iRIGS near GWAS hits, and there might be false positives and false negatives in this set. Therefore, the candidate genes we identified still require thorough experimentation. Second, as with most supervised learning methods, our framework depends on existing patterns of labelled genes, and therefore are less powerful in identifying disease genes with characteristics not well represented in the training set.

Opportunities for future expansion of this strategy include exploring diverse and relevant features and applying better approaches to integrate signals from multiple features. Currently, we chose features from gene expression and biological processes in the prediction. There are other data resources such as cell-type specific gene expression, proteomics and epigenomics data. Using single-cell RNA-sequencing data, it was found that synaptic signaling of upper-layer excitatory neurons and the molecular state of microglia are preferentially affected in ASD [52]. Epigenetics have also been reported to play a role in predisposition to ASD [53]. These data sources could be used in the future expansion of our approach. As IHW takes a single covariate, we took an average of the single feature based predictions to derive an ensemble score for hypothesis weighting for simplicity. We explored other methods that could include multiple dimensions of covariates like AdaFDR [4] and AdaPT [54]. However, our application of AdaFDR did not yield improvements and tend to be less stable; AdaPT takes many iterations of optimization and is computationally expensive as it uses a *p*-value masking procedure. There might be room for further improvement in the way of integrating multiple covariates, which is worthy of future explorations.

## 5. Conclusions

In this paper, we present a three-stage framework to identify risk genes from both GWASs and RVASs: We first obtain training genes in GWAS significant loci via iRIGS, then build machine-learning prediction models to predict each gene’s probability to associate with SCZ using training genes and biological features; and finally we use the prediction scores as informative covariates for hypothesis weighting to improve gene detection power using IHW. We applied the framework to SCZ and ASD gene-based RVASs and observed sizeable improvements on the number of genes discovered. As an increasing volume of contextual information is being generated, we believe that our approach that leverages prediction as covariates in hypothesis weighting provides a valuable contribution to boost statistical significance in RVASs. This approach requires little investment and can be easily applied to the analysis of existing and future studies beyond RVASs.

## Figures and Tables

**Table 1:** Enrichment of top 1000 predicted genes in gene sets implicated in SCZ.

Gene Set ${}^{a}$	OR ${}^{b}$	*p*-Value ${}^{c}$
FMRP-Darnel (832)	14.23	$6.10\times 10^{-249}$
RBFOX1 (556)	11.14	$2.2\times 10^{-140}$
PSD (1444)	5.15	$5.82\times 10^{-126}$
ECG (998)	5.38	$2.28\times 10^{-99}$
PRP (336)	5.06	$4.53\times 10^{-34}$
PRAZ (209)	5.87	$6.90\times 10^{-27}$
NMDAR (59)	18.17	$3.54\times 10^{-24}$
miR-137 targets (281)	4.32	$2.19\times 10^{-22}$
GABA (18)	46.06	$1.07\times 10^{-11}$
SYV (107)	4.32	$7.38\times 10^{-09}$
ARC (25)	13.82	$2.19\times 10^{-08}$
CRF (56)	5.54	$6.81\times 10^{-07}$
mGluR5 (37)	6.28	$2.00\times 10^{-05}$
CCS (73)	3.72	$1.06\times 10^{-04}$

^a^ The numbers of genes in the corresponding gene sets are in parentheses. The source and short description of these gene sets is included in Table A1. ^b^ Odds ratio from one-sided Fisher’s exact test. ^c^ *p*-value from one-sided Fisher’s exact test after Bonferroni correction.

**Table 2:** Number of discoveries from SCZ dataset, by different methods and covariates ${}^{a}$.

Method ${}^{b}$	0.05 ${}^{c}$	0.1	0.2	0.3
IHW-BRAINSPAN	29	34	51	63
IHW-FANTOM5	30	37	54	64
IHW-DEPICT	30	36	57	93
IHW-LAKE	30	35	55	97
IHW-ensemble	30	38	59	134
IHW-shuffled ensemble ^d^	31	31	48	57
BH	31	33	46	64

^a^ At a range of target FDR levels α from 0.05 to 0.3. ^b^ Methods used for *p*-value adjustment. IHWBRAINSPAN/DEPICT/FANTOM5/LAKE/ensemble: Predictions from single data sources (BRAINSPAN, DEPICT, FANTOM5, and LAKE) and ensemble score; BH: Conventional BH-approach. ^c^ α = 0.05. ^d^ Shuffled ensemble: Randomly shuffled the ensemble scores (i.e., “IHW-ensemble”), and used the shuffled scores as the covariate for adjustment.

**Table 3:** Number of discoveries from ASD dataset, by different methods and covariates ${}^{a}$.

Method ${}^{b}$	0.05 ${}^{c}$	0.1	0.2	0.3
IHW-BRAINSPAN	89	142	294	477
IHW-FANTOM5	98	119	207	363
IHW-DEPICT	107	158	329	495
IHW-LAKE	105	149	287	439
IHW-ensemble	112	176	323	658
IHW-shuffled ensemble ^d^	79	95	151	201
BH	76	99	143	199

^a^ At a range of target FDR levels α from 0.05 to 0.3. ^b^ Methods used for *p*-value adjustment. IHWBRAINSPAN/DEPICT/FANTOM5/LAKE/ensemble: Predictions from single data sources (BRAINSPAN, DEPICT, FANTOM5, and LAKE) and ensemble score; BH: Conventional BH-approach. ^c^ α = 0.05. ^d^ Shuffled ensemble: Randomly shuffled the ensemble (i.e., “IHW-ensemble”), and used the shuffled scores as the covariate for adjustment.

## Abbreviations

The following abbreviations are used in this manuscript:

RVAS	rare variant association study
iRIGS	integrative risk gene selector
GWAS	genome-wide associations study
SCZ	schizophrenia
ASD	autism spectrum disorder
FDR	false discovery rate
FWER	family-wise error rate
PTV	protein-truncating variants
MPC	missense badness, PolyPhen-2, and constraint
AUC	area under the receiver-operating characteristic (ROC) curve

## Appendix A

**Table A1:** Gene sets implicated in SCZ.

Gene Set	Short Description
FMRP-Darnel [55]	Fragile X mental retardation (FMRP) protein targets
RBFOX1 [56]	targets of RNA binding protein, fox-1 homolog 1
PSD [57]	post synaptic genes
ECG [58]	evolutionary constrained genes
PRP [59]	genes related to presynaptic proteins
PRAZ [59]	genes in the presynaptic active zone
NMDAR [35]	components of the N-methyl-D-aspartate (NMDA) network
miR-137 targets [60]	miRNA-137 targets
GABA [61]	components of the GABA receptor complex
SYV [59]	synaptic vesicles
ARC	neuronal activity-regulated cytoskeleton-associated proteins
CRF [62]	chromatin remodeling factors
mGluR5 [46]	components of the metabotropic glutamate receptor 5 complex
CCS [63]	calcium channel and signaling genes
